# Gene expression profiles in mesenchymal stromal cells from bone marrow, adipose tissue and lung tissue of COPD patients and controls

**DOI:** 10.1186/s12931-023-02314-8

**Published:** 2023-01-21

**Authors:** Dennis Kruk, Anna C. Y. Yeung, Alen Faiz, Nick H. T. ten Hacken, Wim Timens, Toin H. van Kuppevelt, Willeke Daamen, Danique Hof, Martin C. Harmsen, Mauricio Rojas, Irene H. Heijink

**Affiliations:** 1 Department of Pathology and Medical Biology, University of Groningen, University Medical Center Groningen, Hanzeplein 1, 9713 GZ Groningen, Groningen The Netherlands; 2 Groningen Research Institute for Asthma and COPD, University of Groningen, University Medical Center Groningen, Groningen, The Netherlands; 3 Respiratory Bioinformatics and Molecular Biology (RBMB) Group, The University of Technology Sydney, Ultimo, NSW Australia; 4 Woolcock Institute of Medical Research, The University of Sydney, Glebe, NSW Australia; 5 Department of Pulmonary Diseases, University of Groningen, University Medical Center Groningen, Groningen, The Netherlands; 6 Department of Biochemistry, University of Nijmegen, Radboud University Medical Center, Nijmegen, The Netherlands; 7 Division of Pulmonary, Critical Care and Sleep Medicine, The Ohio State University, Columbus, OH USA

**Keywords:** COPD pathology, Mesenchymal stromal cells, Lung tissue, Bone marrow, Adipose tissue, Transcriptomics

## Abstract

**Background:**

Chronic obstructive pulmonary disease (COPD) is characterized by irreversible lung tissue damage. Novel regenerative strategies are urgently awaited. Cultured mesenchymal stem/stromal cells (MSCs) have shown promising results in experimental models of COPD, but differences between sources may impact on their potential use in therapeutic strategies in patients.

**Aim:**

To assess the transcriptome of lung-derived MSCs (LMSCs), bone marrow-derived MSCs (BM-MSC) and adipose-derived MSCs (AD-MSCs) from COPD patients and non-COPD controls.

**Methods:**

We studied differences in gene expression profiles between the MSC-subtypes, as well as between COPD and control using RNA sequencing (RNA-seq).

**Results:**

We show that besides heterogeneity between donors, MSCs from different sources have strongly divergent gene signatures. The growth factors *FGF10* and *HGF* were predominantly expressed in LMSCs. MSCs from all sources displayed altered expression profiles in COPD, with most pronounced significantly up- and downregulated genes in MSCs from adipose tissue. Pathway analysis revealed that the most differentially expressed genes in COPD-derived AD-MSCs are involved in extracellular matrix (ECM) binding and expression. In LMSCs, the gene that differed most strongly between COPD and control was *CSGALNACT1*, an ECM modulating gene.

**Conclusion:**

Autologous MSCs from COPD patients display abnormalities with respect to their transcriptome, which were surprisingly most profound in MSCs from extrapulmonary sources. LMSCs may be optimally equipped for lung tissue repair because of the expression of specific growth factor genes.

**Supplementary Information:**

The online version contains supplementary material available at 10.1186/s12931-023-02314-8.

## Introduction

Chronic Obstructive Pulmonary Disease (COPD) is a chronic inflammatory lung disease, according to WHO the third leading cause of death worldwide [1]. Primary risk factors for COPD include inhalation of noxious particles, such as cigarette smoke and air pollutants, leading to chronic inflammation in the lungs, lung tissue damage and aberrant tissue repair in COPD patients. The disease is characterized by excess mucus secretion (chronic bronchitis), (small) airway wall thickening and destruction of the alveoli (emphysema), leading to airflow limitation and accelerated lung function decline. The loss of alveolar septa is irreversible and cannot be treated with current therapies. Therefore, there is an urgent need for novel treatments strategies to combat the progressive loss of lung function by reinforcing alveolar repair mechanisms, including regenerative medicine approaches.

Cell-based strategies have shown promising results in immune-mediated diseases and in experimental models of COPD and emphysema [2, 3]. The most widely used stem cell population for therapeutic application in pre-clinical and clinical studies is the mesenchymal stromal/stem cell (MSC). MSCs are multipotent stem cells that can be derived from various stromal tissues, including bone marrow, adipose tissue and lungs [4]. The beneficial effects of MSCs have been mainly attributed to paracrine mechanisms, secreting regenerative growth factors as well as anti-inflammatory/immunosuppressive factors [3]. Their use has been widely evaluated for improvement of lung function in animal models of emphysema, leading to reduced inflammation while supporting repair of alveolar damage and restoring lung structure [2]. However, whereas human clinical trials demonstrated the therapy’s safety, treatment with autologous bone marrow-derived MSCs (BM-MSCs) has not yet resulted in restoration of alveolar structure nor beneficial effects on lung function [5]. This may be due to a variety of factors, including lack of insight into optimal route of administration, dosing, source, timing and frequency of treatment and limited retention of MSCs. It is unknown whether intravenously administered BM-MSCs are equipped to engraft and survive in lung tissue. In fact, due to lack of standardization of protocols and limited knowledge on the properties of lung resident MSCs, it is unknown which source of MSCs is suited to realize regenerative effects in the lung. While MSCs from different sources share common features, such as the secretion of regenerative and anti-inflammatory factors, expression profiles may differ. Previous reports have shown differences in the effectiveness of MSCs from different types of tissue to reduce manifestations of COPD in animal models. For example, when comparing the efficacy of lung-derived MSCs (LMSCs) and BM-MSC, cells from both sources ameliorated lung damage, although LMSCs showed higher expression of specific endothelial adhesion molecules and higher retention in the lungs [6]. In a study comparing LMSCs, BM-MSCs and adipose-derived MSCs (AD-MSCs), BM-MSCs displayed most pronounced beneficial systemic effects, while AD-MSCs and LMSCs achieved more significant reduction in fractional area of alveolar collapse [4].

One of the challenges using autologous MSCs may be that cells isolated from a diseased microenvironment, with chronic inflammation, a high burden of oxidative stress and extensive tissue destruction, may display impairments in their regenerative capacity. Since COPD is a systemic disease often accompanied with metabolic abnormalities, cells from extrapulmonary tissues may also be affected. Because of the high plasticity of MSCs, their functions may additionally be changed upon in vitro culturing. It is therefore particularly relevant to compare MSCs that have been cultured in the exactly the same way.

Together, in addition to the profiling of MSCs from the lung, questions that remain are whether MSCs from COPD lungs display abnormalities in their gene signature and to what extent abnormalities can be found in MSCs from extrapulmonary sources in COPD patients. Therefore, the aim of this study was to assess the transcriptome of LMSCs, BM-MSCs and AD-MSC from COPD patients and non-COPD controls. We studied differences in gene expression profiles between the MSC-subtypes, as well as between COPD and control using RNA sequencing (RNA-seq).

## Methods

### Subjects

Parenchymal lung tissue was left-over material derived from 7 emphysema patients with GOLD stage III-IV COPD undergoing lung transplantation, tumor resection or lung volume reduction surgery and from leftover lung material from 7 non-COPD controls undergoing tumor resection surgery. Lung tissue was collected distant from the tumor and checked for abnormalities by an experienced pathologist and if indicated excluded from our study. Subcutaneous adipose tissue was collected from 4 emphysema patients undergoing bronchoscopic lung volume reduction surgery (from 3 of these we also collected lung tissue), lung cancer surgery, tumor resection surgery or lung transplantation and 3 non-COPD controls undergoing tumor resection surgery (from 1 of these we also collected lung tissue). The study protocol was consistent with the Research Code of the University Medical Center Groningen (https://umcgresearch.org/en-GB/w/research-code-umcg) and national ethical and professional guidelines (https://www.coreon.org). Bone marrow was collected from vertebrate discs of 7 COPD patients and 7 non-COPD controls (University of Pittsburgh School of Medicine, Pittsburgh, Pennsylvania). See Table 1 for patient characteristics.

**Table 1:** Characteristics of subjects included in the study

	LMSCs		AD-MSCs		BM-MSCs	
	Control (n = 7)	COPD (n = 7)	Control (n = 3)	COPD (n = 4)	Control (n = 7)	COPD (n = 7)
Gender	4 F / 3 M	5 F / 2 M	1 F / 2 M	3 F / 1 M	1 F / 6 M	2 F / 5 M
Smoking history						
Current	14%	14%	33%	0%	Unknown	Unknown
Former	28%	86%	33%	100%	Unknown	Unknown
Never	43%	0%	33%	0%	Unknown	Unknown
Age [years]	67 (41–74)	61 (45–74)	66 (41–69)	61 (45–66)	51 (43–57)	51 (44–55)
FEV1%Pred	92 (71–110)	19 (13–49)	109 (92–115)	18,5 (13–24)	Unknown	Unknown
FEV1/FVC	78 (66–81)	27 (22–24)	79 (69–80)	23,5 (22–27)	Unknown	Unknown

FEV1%Pred = Predicted value for Forced Expiratory Volume in 1 s; FEV1 = Forced Expiratory Volume in 1 s; FVC = Forced Vital Capacity. For age, FEV1%Pred and FEV1/FVC, group medians with ranges are shown. For bone marrow donors, the diagnosis of COPD was confirmed by CT scan and histology. Exclusion criteria for subject inclusion in the study were the diagnosis of asthma, indications of lung infection, COPD GOLD stage classification of I or II, alpha-1 antitrypsin deficiency or abnormalities in tissue structure

### Cell isolation and culture

LMSCs were acquired from ~ 5 mm^2^ blocks of peripheral parenchymal lung tissue as described in the Additional file 1. AD-MSCs were isolated from 1 cm^3^ cubes of subcutaneous adipose tissue and BM-MSCs we isolated from vertebrate discs as described in the Additional file 1.

After defrosting, LMSCs, AD-MSCs and BM-MSCs were grown to confluence, plated in 6 wells plates and cultured for 2–3 days in low-glucose DMEM with 10% fetal calf serum (FCS), 1% L-glutamine, 1% Penicillin–Streptomycin (Gibco) to ~ 90% confluence, serum-deprived overnight and placed into fresh serum free medium for 24 h. Cells were collected and lysed in TRI reagent (MRC, Cincinnati, OH) for RNA isolation. MSC surface markers were expressed by isolated populations as assessed by flow cytometry and described previously [7], in accordance with the criteria of the International Society for Cellular Therapy for characterization [8].

### RNA generation and isolation

Total mRNA was extracted from cultured MSCs using a chloroform method, followed by an additional clean-up step using the RNeasy MinElute Cleanup Kit (Qiagen, Venlo, Netherlands) in accordance with the manufacturer’s instructions. RNA quality and quantity were checked using spectrophotometric (Nanodrop, ThermoScientific, Waltham, MA) and microfluidic methods (RNA 6000 Nano chip, 2100 Bioanalyzer, Agilent Technologies, Milan, Italy).

### Illumina library prep and sequencing run

All RNA samples used for library prep had an RNA integrity number (RIN) value above 7.8. Ribosomal RNA was removed by NEXTflex^®^ Poly(A) beads (Bio Scientific) depletion following the manufacturer’s instructions. Purified RNA was then used with the NEXTflex Rapid Directional qRNA-Seq Kits (Bio Scientific) to generate the library according to the manufacturer’s instructions as outlined in the Additional file 1.

### Data analysis

RNAseq data were analyzed using the R-package EdgeR (3.28.1). A linear model was conducted correcting for gender and age to compare between COPD and control samples within each cell type, as well as a comparison across cell types regardless of disease state.

### Single-cell expression profile

Cell type clustering of a single-cell atlas of nasal, airway and lung samples was visualized as a uniform manifold approximation and projection (UMAP) using an interactive web portal of the Human Lung Cell Atlas [9]. The visualization was then focused on a signature of the 5 genes found to be specific to LM cells.

### Cellular deconvolution

Cellular deconvolution of bulk RNA-seq data was performed to estimate the proportions of different cell types from the gene expression for all bulk RNA-Seq datasets [10], as described in the Additional file 1.

### Single cell U-maps

Single cell u-maps obtained from bronchial biopsies were accessed through the Sanger single cell online portal (https://asthma.cellgeni.sanger.ac.uk).

### Pathway analysis

Pathway analysis was performed using gProfiler (version e95_eg42_p13) on the significant gene list or the top 200 genes if the list exceeds 200 genes.

### qPCR

Cells were lysed in TRI reagent (MRC, Cincinnati, OH) for RNA isolation using the chloroform extraction method. cDNA synthesis (iScript cDNA synthesis kit (BioRad, Hercules, CA) and qPCR analysis using TaqMan (Life Technologies, Waltham, MA) were performed in accordance to the manufacturer’s instructions. Validated TaqMan probes were used for the assessment of expression of the housekeeping gene *B2M* and *PPIA* and the epithelial growth factors *FGF10*, *HGF*, *FGF7/KGF* and *CSGALNACT1* in technical duplicates.

### Seeding of decellularized scaffolds with LMSCs

Decellularized lung tissue scaffolds were generated from 3 GOLD stage IV COPD patients with emphysema and 3 non-emphysema controls. Lung tissue blocks (~ 3 cm^3^) were decellularized and reseeded with LMSCs as described in the Additional file 1. Paraffin sections were processed and stained with the single chain variable fragment antibody IO3H10 for detection of chondroitin sulfates [11].

### Statistics

The Mann Whitney U test was used when testing for differences between two groups.

## Results

### Highly divergent gene expression profiles between MSCs from different sources

**Fig. 1:**
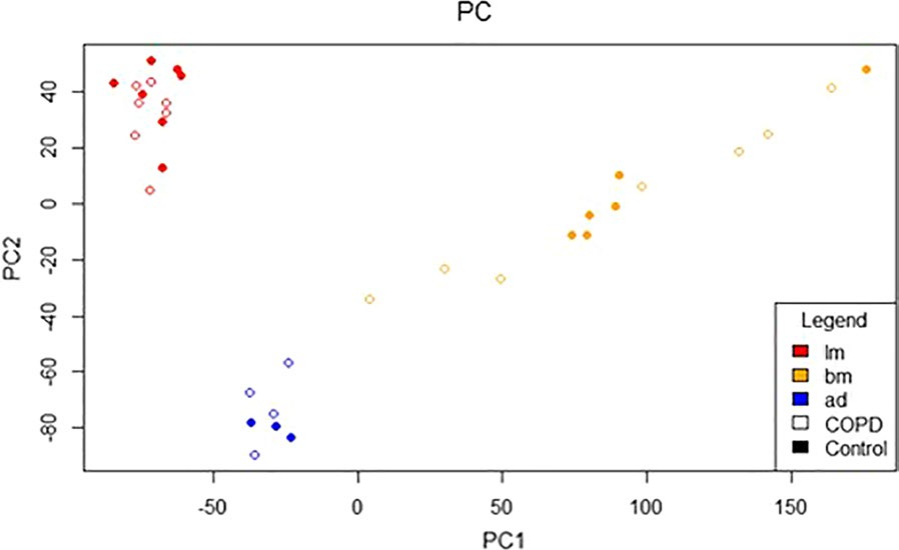
Principal component (PC) analysis. PC in the RNA obtained from lung-derived MSCs (LM; red), bone marrow-derived MSCs (BM; yellow) and adipose-derived MSCs (AD; blue) from COPD patients (open circles) and controls (closed circles)

We first compared gene expression profiles between the different sources of MSCs. MSCs from the different sources were cultured under the same conditions to ensure that differences between the sources were not caused by differences in culture conditions. Principal component analysis shows clustering of MSCs from controls and COPD patients of the three different sources (Fig. 1), indicating distinct expression profiles, which are not driven by COPD.

**Fig. 2:**
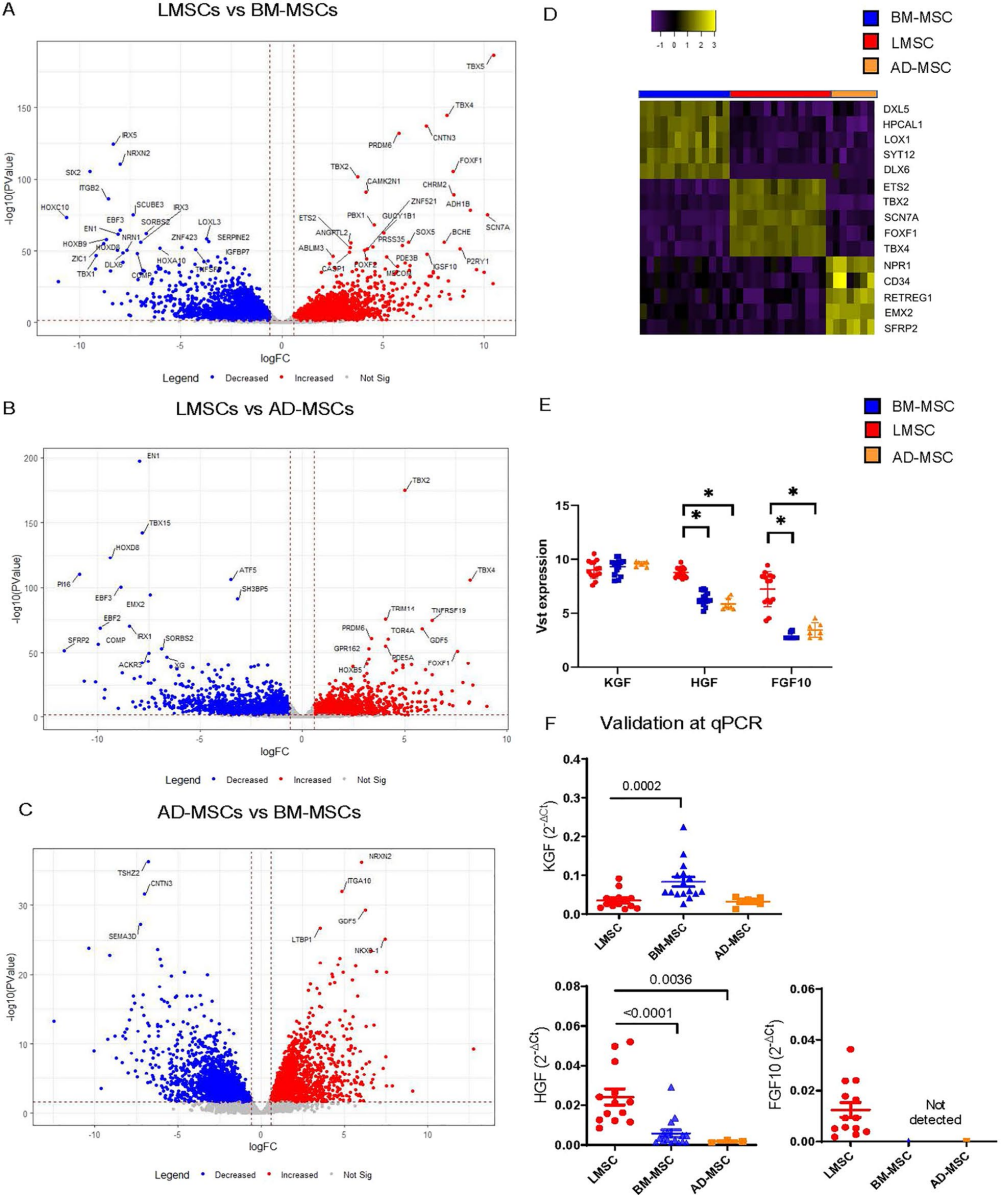
Differential gene expression in MSCs from different sources. Lung-derived MSCs (LMSCs), bone marrow-derived MSCs (BM-MSCs) and adipose-derived MSCs (AD-MSCs) from COPD patients and controls were seeded in duplicates, grown to confluence and serum deprived overnight. Cells were harvested after 24 h, RNA was isolated and processed for RNA sequencing. Volcano plots demonstrate differentially expressed genes between the cell types (COPD and control groups combined). Blue represents genes significantly lower expressed; red represents genes significantly higher expressed. False discovery rate (FDR) < 0.05 was used as cut off*.* **A** LMSCs vs BM-MSCs. **B** LMSCs vs AD-MSCs. **C** BM-MSCs vs AD-MSCs. **D** Heatmap showing gene sets with specific expression in each cell source*.* Specific gene expression profile of each source after variance stabilizing transformation (Vst). **E** Comparison of the expression of *KGF, FGF10* an*d HGF* between the different subsets. Medians ± interquartile range (IQR) are shown. **F** mRNA expression of growth factors *KGF, FGF10* and *HGF* was assessed by qPCR and normalized for housekeeping gene *B2M* and expressed as 2^−ΔCt^. Medians are indicated. Statistical significance was determined using the Mann–Whitney U test. P values are as indicated. * = P < 0.05 between the indicated values. P < 0.05 was considered statistically significant

When comparing gene expression between LMSCs vs BM-MSCs, LMSCs vs AD-MSCs and AD-MSC vs BM-MSCs (COPD and control groups combined), we observed strongly divergent gene expression profiles. In total 8746 genes were differentially expressed between LMSCs and BM-MSCs, 3501 genes between LMSCs and AD-MSCs, and 7482 genes between BM-MSCs and AD-MSCs. We observed a specific LMSC gene signature of *ETS2*, *TBX5*, *SCN7A*, *FOXF1* and *TBX4* (Fig. 2A–D). In addition to *FOXF1,* previously *HOXB5* and *SFRP1* have been identified as lung-specific genes [12]*.* When assessing differences in expression of these genes between LMSCs and BM-MSCs, we found significantly higher expression of both genes in LMSCs (False discovery rate (FDR)-corrected P value = 2.00E-11 and 8.43E-36 for *HOXB5* and *SFRP1* respectively). Furthermore, since especially the expression of regenerative factors is of relevance with respect to therapeutic effects in emphysema, we performed targeted analysis on growth factors that play a critical role in alveolar epithelial regeneration based on literature, FGF7/KGF, FGF10 and HGF [13–18]. We observed that *FGF10* and *HGF* were much more strongly expressed in LMSCs compared to the other sources, while *KGF* was also strongly expressed by BM-MSCs and AD-MSCs with significantly higher expression in MSCs from these sources compared to LMSCs (Fig. 2E). This was confirmed by qPCR (Fig. 2F).

### Cells with an overlapping gene expression profiles as LMSCs are present in human lungs

Next, we assessed if cell clusters reside in human lung tissue with an overlapping gene expression profile as in vitro-cultured LMSCs. We used an existing dataset of single cell RNA sequencing in peripheral human lung tissue [9]. The annotation of MSCs in single cell datasets is rare, but we observed that a specific cluster of (myo)fibroblasts-like cells expresses the gene signature of cultured LMSCs, based on a composite score of the 5 signature genes (Additional file 1).

### Differences in gene expression between COPD and control are more pronounced in MSCs from bone marrow and adipose tissue than from lungs

**Fig. 3:**
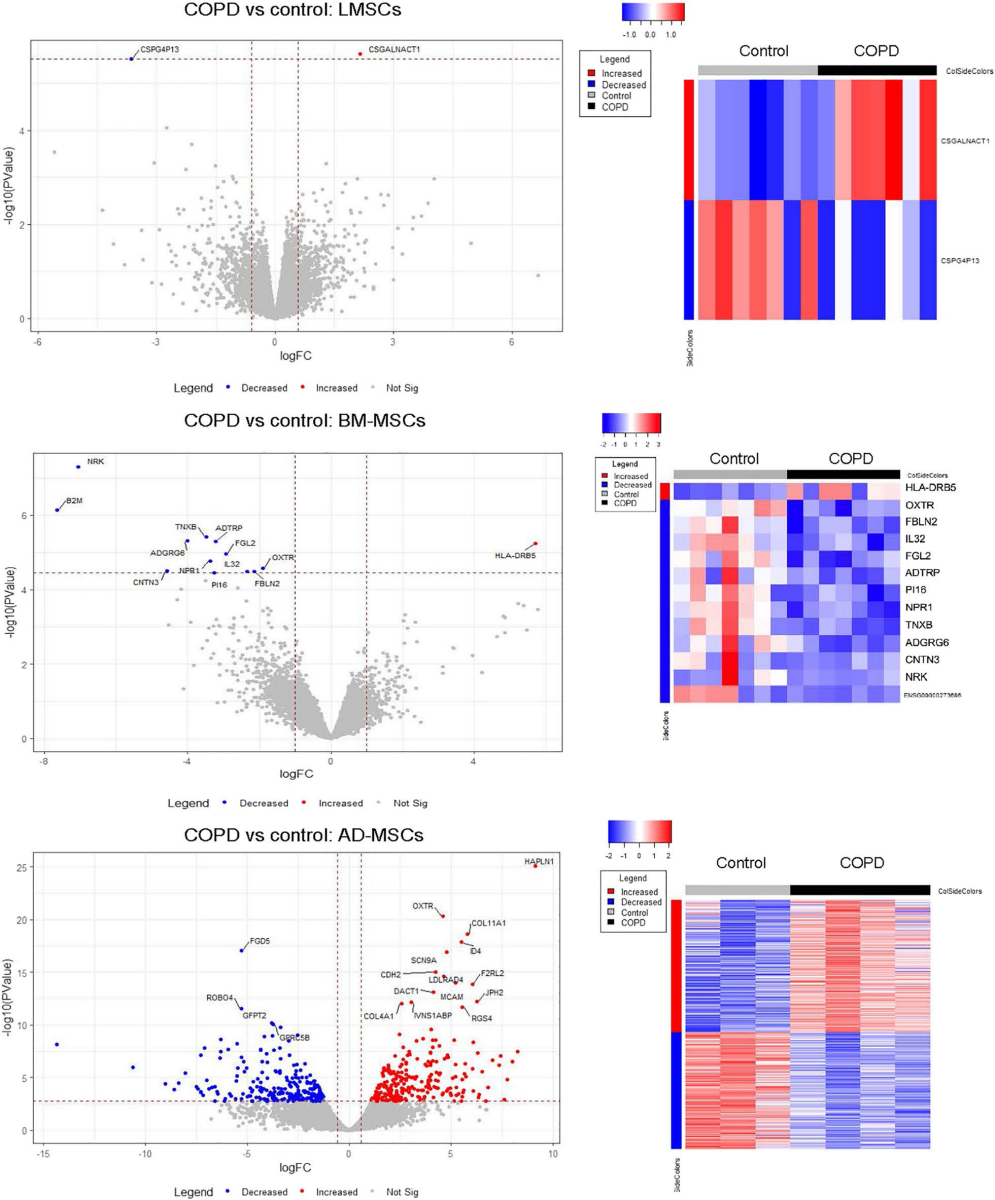
Differential gene expression between COPD and control in MSCs from different sources. Lung-derived MSCs (LMSCs), bone marrow-derived MSCs (BM-MSCs) and adipose-derived MSCs (AD-MSCs) from COPD patients and controls were seeded in duplicates, grown to confluence and serum deprived overnight. Cells were harvested after 24 h, RNA was isolated and processed for RNA sequencing was performed to compare gene expression profiles between COPD and control. Volcano plots demonstrate differentially expressed genes between COPD and control groups. Blue represents significant genes significantly lower and red represents genes significantly higher expressed in COPD-derived cells vs control-derived cells. False discovery rate (FDR) < 0.05 was used as cut off. The right panels show heatmaps

In order to assess whether COPD-derived MSCs display abnormalities and whether these are intrinsic or related to the diseased lung microenvironment, we compared non-COPD control and COPD-derived profiles for all sources (Fig. 3). Unexpectedly, we observed that only 2 genes were differently expressed between the LMSCs from severe, emphysematous COPD patients and controls (1 lower, 1 higher expressed), while 13 genes were differently expressed in COPD-derived vs control-derived BM-MSCs (12 lower, 1 higher expressed). The higher expressed gene was *HLA-DRB5*, encoding the major histocompatibility complex (MHC) region DRB5. The most strongly downregulated gene was *NRK*, encoding Nik-related kinase, a Ser/Thr kinase involved in developmental processes. Especially AD-MSCs showed strong differences in gene expression profiles between severe, emphysematous COPD and control, with many up- and downregulated genes. The upregulated genes in COPD-derived AD-MSCs included pericyte markers *CDH2* and *COL4A1*. Pathway analysis revealed that the most downregulated genes in COPD-derived AD-MSCs are involved in binding to specific components of the extracellular matrix and growth factors, while the most upregulated genes are involved in extracellular matrix expression and developmental pathways (See Tables 2 and 3 for the top 10 most downregulated and upregulated pathways respectively).

**Table 2:** Pathway analysis of significantly decreased genes in AD-MSCs, COPD vs control

Source	term_name	term_id	adjusted_p_value
GO:MF	Heparin binding	GO:0008201	0.000161618
GO:MF	Glycosaminoglycan binding	GO:0005539	0.000876765
GO:MF	Signaling receptor binding	GO:0005102	0.001008396
GO:MF	Sulfur compound binding	GO:1901681	0.001657873
GO:MF	Fibronectin binding	GO:0001968	0.003329845
GO:MF	Insulin-like growth factor binding	GO:0005520	0.021832888
GO:MF	Platelet-derived growth factor-activated receptor activity	GO:0005017	0.0291196
GO:MF	Molecular transducer activity	GO:0060089	0.039974476
GO:BP	Negative regulation of response to wounding	GO:1903035	0.000141464
GO:BP	Regulation of cell communication	GO:0010646	0.000257333

**Table 3:** Pathway analysis of significantly increased genes in AD-MSCs, COPD vs control

Source	term_name	term_id	adjusted_p_value
GO:MF	Extracellular matrix structural constituent	GO:0005201	7.89E-07
GO:MF	Extracellular matrix binding	GO:0050840	0.000773464
GO:MF	Extracellular matrix structural constituent conferring tensile strength	GO:0030020	0.011540193
GO:MF	Sodium channel activity	GO:0005272	0.012683475
GO:BP	Anatomical structure morphogenesis	GO:0009653	2.41E-11
GO:BP	Circulatory system development	GO:0072359	9.99E-08
GO:BP	Anatomical structure development	GO:0048856	2.86E-07
GO:BP	Multicellular organism development	GO:0007275	6.54E-07
GO:BP	Developmental process	GO:0032502	1.84193E-06
GO:BP	Forebrain development	GO:0030900	3.93749E-06

The most and only significantly upregulated gene in COPD-derived LMSCs was *CSGALNACT1*, a gene encoding the enzyme that initiates chondroitin sulfate (CS) biosynthesis The only downregulated gene in COPD-derived LMSCs was *CSPG4P13*, chondroitin sulfate proteoglycan 4 pseudogene 13, a non-functional gene.

**Fig. 4:**
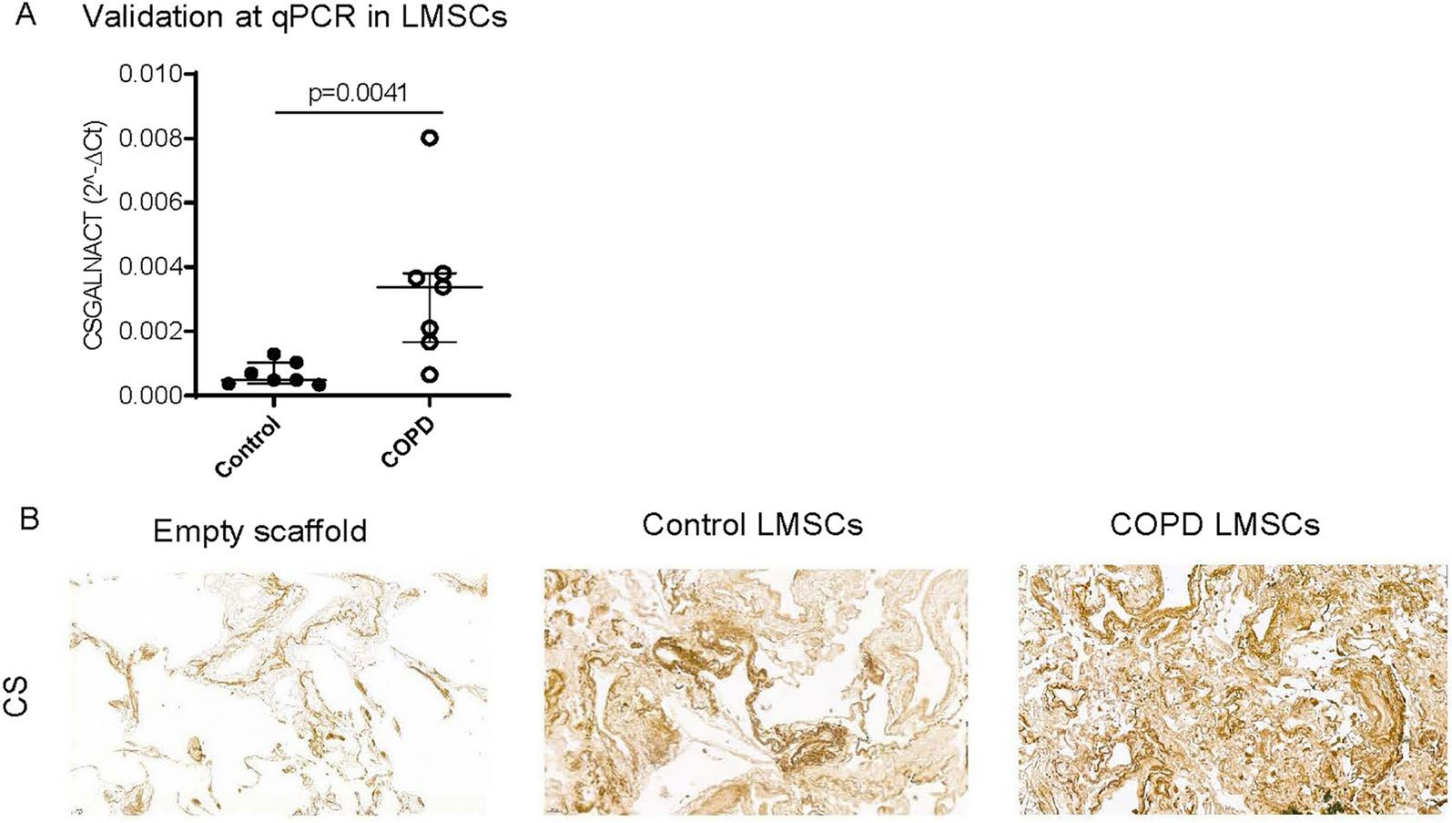
Differential expression of *CSGALNACT1* between lung-derived MSCs (LMSCs) from COPD and control. **A** LMSCs from COPD patients and controls were seeded in duplicates, grown to confluence and serum deprived overnight. Cells were harvested after 24 h and RNA was isolated. mRNA expression of *CSGALNACT1* was assessed by qPCR and normalized for housekeeping gene *B2M* and expressed as 2^−ΔCt^. Medians are indicated. Statistical significance was determined using the Mann–Whitney U test. The P values are indicated. P < 0.05 was considered statistically significant. **B** Decellularized emphysematous tissue lung scaffolds were reseeded with/without COPD or control-derived LMSCs and cultured for 2 weeks. Scaffolds without cells were treated identically to the cell-seeded scaffolds. Paraffin sections were prepared and stained for chondroitin sulfates (CS). Representative images are shown

The higher expression of *CSGALNACT1* was confirmed by qPCR (Fig. 4A). Staining of decellularized lung tissue scaffolds that were reseeded with LMSCs confirmed that the gene is actively translated in LMSCs, as the intensity of chondroitin sulfate staining was stronger in reseeded compared to unseeded scaffolds (Fig. 4B). The intensity was highly variable and chondroitin sulfates were also present in the unseeded scaffolds. Therefore, we were unable to properly quantify potential differences between the scaffolds seeded with COPD and control-derived LMSCs.

## Discussion

In this study, we compared gene expression profiles of MSCs derived from lung, bone marrow and adipose tissue of COPD patients and non-COPD controls. MSCs from each source had a specific gene signature. Comparing COPD to control, the only genome-wide significantly different expressed gene in LMSCs was *CSGALNACT1,* while higher number of differentially expressed genes were observed in BM-MSCs and AD-MSCs.

The differences between COPD and control in MSCs from extrapulmonary tissues may reflect systemic effects of either smoking or the disease. Of note, we did not correct for presence of tumors in the lung tissue, which may have had effects systemic effects as well. The different signatures of MSCs from lung tissue versus other sources and between COPD and control should be taken into account when considering MSCs for therapeutic strategies in emphysema, the latter especially when using autologous MSCs. As for the tissue specificity, a specific profile of regenerative factors may be needed to realize tissue regeneration in each specific organ. For instance, HGF and FGF10 are known to mediate alveolar repair and stimulate proliferation of alveolar epithelial progenitors [14–16, 19]. Notably, we previously observed that both factors are lower expressed in COPD-derived LMSCs compared to those from controls [20]. Here, we show that genes encoding both growth factors are predominantly expressed in MSCs from the lung. Rolandsson and co-workers also observed important differences between LMSCs and BM-MSCs using a microarray [12]. Their study was the first to confirm that lung and bone marrow resident MSCs possess tissue specific properties. Although LMSCs had a higher colony forming capacity and lower osteogenic differentiation potential, the authors observed an overall more similar gene expression pattern in LMSCs and BM-MSCs compared to our study, with 89 genes differently expressed. Similar to our study, MSCs from lung and bone marrow were from different donors using the same culture protocol, whereas the isolation and culture protocols differ between our studies. Further, we isolated LMSCs from explanted peripheral lung tissue, while Rolandsson and co-workers used transbronchial biopsies in live patients, although it is unclear how this would explain the higher similarity between MSCs from different sources. In line with our findings, Rolandsson and co-workers showed that *FOXF1* as well as *HOXB5* and *SFRP1* were amongst the lung-specific genes. All these genes have been demonstrated crucial for human lung development and branching [21, 22]. We observed that signature genes of LMSCs include *FOXF1, TBX2, TBX4, SCN7A* and *ETS2,* and that a stromal cell subset exists in lung tissue in vivo with a similar expression profile. Of interest, forkhead box F1 (FOXF1) is a lung embryonic mesenchyme-specific transcription factor with persistent expression into adulthood in mesenchymal stromal cells [23]. In murine studies, *Foxf1* + cells were shown to encompass a stem cell subset of collagen 1-expressing mesenchymal cells with clonogenic potential and capacity to generate lung epithelial organoids [24]. Interactions between FOXF1 and sonic hedgehog (SHH), T-box transcription factor (TBX4), TBX2 and FGF10 pathways have been described, proposing an essential transcriptional network during early lung organogenesis [25]. *SCN7A* encodes an atypical sodium channel. It has been identified as signature gene of the stromal tumor micro-environment associated with survival of lung cancer [26] and is expressed by alveolar fibroblasts [27]*.* Ets2 a ubiquitous transcription factor that is induced by HGF-MET signaling and is activated after phosphorylation at threonine-72 [28]. Previous studies highlighted the importance of phosphorylated Ets2 in lung inflammation and extracellular matrix remodeling, pathways involved in pulmonary fibrosis [29]. It will be of interest to further study the role of these LMSC signatures genes in lung tissue regenerative processes.

Strikingly, the differences between COPD and control were most pronounced in AD-MSCs, followed by BM-MSCs, and the lowest number of differentially expressed genes was found in LMSCs. So far, clinical studies in COPD using cell-based strategies have focused on autologous BM-MSCs. We observed that the top-hit gene upregulated in COPD-derived BM-MSCs was *HLA-DRB,* encoding MHC region DRB5. Genetic variation in this gene has been associated with interstitial lung disease [30] and with circulating levels of IL-6 [31], a pro-inflammatory cytokine with higher levels in COPD. As for AD-MSCs, the pathways differently expressed between cells from emphysema patients and controls suggest abnormalities in extracellular matrix-growth factor binding, and may thus reflect impaired adhesion/migration responses. The extent of differentially expressed genes in AD-MSCs may be a consequence of metabolic alterations that have been associated with COPD, although caution needs to be taken given the small sample number of AD-MSC donors in our study. Despite this, it is tempting to speculate on the implications of observed abnormalities in native AD-MSCs in COPD. To the best of our knowledge, it is unknown whether AD-MSCs from subcutaneous adipose tissue in the thoracic cavity have the potential to migrate into the lung tissue upon injury. The ability to differentiate towards adipocytes/adipocyte-like cells could be of relevance, as adipocytes highly resemble lipofibroblasts, which are well known to support regenerative processes [32]. The highest upregulated gene in AD-MSCs from COPD patients was *HAPLN1*, encoding hyaluronan and proteoglycan link protein 1. *HAPLN1* is known to be expressed in lung fibroblasts, stabilizing aggregates of proteoglycan monomers with hyaluronic acid in the ECM, which can lead to fibrotic remodeling [33]. Collectively, differences between COPD and control-derived BM-MSCs and AD-MSCs may be of relevance when considering autologous MSCs for the treatment of COPD. Notably, we should also take into account that MSCs may change their phenotype upon administration.

It was somewhat surprising to find only 2 genes with genome-wide significance to be differently expressed between the lung-derived cells from emphysema and control donors. This may reflect absence of major differences between emphysema and control-derived LMSCs, at least in these specific subjects, but may also be due to the loss of a COPD-specific phenotype upon in vitro expansion, although this was apparently not the case for AD-MSCs and BM-MSCs. As mentioned earlier, we previously observed differences in several read-outs between LMSCs from COPD patients and controls [7]. The difference between our two studies is that LMSCs were previously grown in high-glucose media (25 mM), while here we used low-glucose media (5.5 mM) in order to be able to compare to the MSCs from the other sources. Future studies will have to reveal whether a low-glucose (normal) environment can normalize defects observed in LMSCs from COPD.

The most strongly upregulated gene in LMSCs from emphysema patients was *CSGALNACT1*, which encodes chondroitin sulfate N-acetylgalactosaminyltransferase-1 (CSGalNAcT-1). This enzyme initiates the biosynthesis of chondroitin sulfate chain biosynthesis on the so-called GAG-protein linker region tetrasaccharide. Subsequently, this can lead to and dermatan sulfate biosynthesis. Although the functional consequences of high *CSGALNACT1* expression of need further investigation, our data suggest that LMSCs can modulate the ECM in their micro-environment, resulting in higher chondroitin and/or dermatan sulfate ratios and as consequence potentially lower heparan sulfate ratios. Of interest, lower levels of heparan sulfate proteoglycans have been observed in COPD lung tissue [34]. Proteoglycans bind growth factors and thus instruct cellular attachment, proliferation and differentiation. Specifically, heparan sulfates act as co-factors to enhance FGF10 signaling [35], thereby potentially supporting alveolar epithelial activation as well as mobilization and recruitment of lung-resident MSCs [36].

The most strongly downregulated gene in emphysema-derived LMSCs was a pseudogene, *CSPG4P13*. Pseudogenes can act as decoy for microRNAs, potentially enhancing the expression of their respective genes, but to the best of our knowledge this has not been described for CSPG4P13. The protein encoded by *CSPG4,* chondroitin sulfate proteoglycan 4, is a well-known marker for pericytes, but further investigation has to show the potential consequences of lower *CSP4P13* expression in LMSCs.

A limitation of our studies is that the translation to protein data needs to be largely confirmed, as previously done for lower HGF and decorin levels in LMSCs from COPD patients versus controls [20]. We did perform staining for chrondroitin sulfate in decellularized scaffolds reseeded with LMSCs, confirming their ability to modify the ECM. However, no differences were readily apparent visually between scaffolds re-seeded with COPD and control-derived LMSCs and quantification was challenging given the presence of CS on empty scaffolds. Therefore, further functional studies will be required in order to confirm the differences between COPD and control derived MSCs.

Together, our data suggest that for cell-based strategies using MSCs, the differences in gene expression profiles between MSCs from different sources should be taken into consideration. LMSCs may be optimally equipped for lung tissue repair because of the expression of specific growth factor genes. Autologous MSCs from COPD patients may show abnormal regenerative responses, even or especially when cells from extrapulmonary sources are considered.

## Supplementary Information

**Additional file 1.** Online data supplement.

## Data Availability

The summary statistics of the performed analyses are included in the additional files of the published article. The datasets used during the current study are available from the corresponding authors on reasonable request.
