# Supplementary material for: Gene expression profiles in mesenchymal stromal cells from bone marrow, adipose tissue and lung tissue of COPD patients and controls
Source: Respir Res. 2023 Jan 21;24:22. doi: 10.1186/s12931-023-02314-8 (PMC9863276; doi:10.1186/s12931-023-02314-8)
Supplement: Supplementary file 1 — Additional file 1. Online data supplement. [file 12931_2023_2314_MOESM1_ESM.docx]

**Gene expression profiles in mesenchymal stromal cells from bone marrow, adipose tissue and lung tissue of COPD patients and controls**

Dennis Kruk, Anna Yeung, Alen Faiz, Nick ten Hacken, Wim Timens, Toin H. van Kuppevelt, Willeke Daamen, Danique Hof, Martin C. Harmsen, Mauricio Rojas, Irene Heijink

*Online data supplement*

**Methods**

**Cell isolation and culture**

Tissue was dissected and treated with 0.25% trypsin-EDTA solution (Gibco) for 15 min after which tissue sections transferred to 6-well plates coated with 30 µg/ml fibronectin and 10 µg/ml BSA (Sigma-Aldrich, Saint Louis, MO). Sections were covered with 1 ml per well culture medium (DMEM/F12 medium (Gibco) containing 1% Glutamax, 10% FCS, and 100 U penicillin with 100 µg/ml streptomycin (Gibco, 1%P/S). Cells were grown out for 2-3 weeks as plastic adherent cells. At approximately 30% confluence, tissue sections were removed and cells were passaged to uncoated plates and cultured to ~90% confluence. Cells were stored in liquid nitrogen before use after expansion for 1 additional passage. In passage 2, cells were seeded in 6-well plates to assess the expression MSC surface markers by flow cytometry in accordance with the criteria of the International Society for Cellular Therapy for characterization as described[1], which were expressed by isolated populations as described previously[2].

AD-MSCs were isolated from 1 cm^3^ cube of subcutaneous adipose tissue. Tissue was first washed in HBSS, then manually minced into small pieces, washed in PBS + 1% BSA multiple times and then dissociated in PBS + 1% BSA + 0.1% collagenase for 1.5 h at 37 °C. The cellular suspension was then filtered over a 0.4 µm filter, the fat removed and the cell pellet washed several times with PBS + 1% BSA. Red blood cells were lysed and leftover cells plated out in a 6-well plate. Cells were expanded for 1 additional passage before cells were frozen.

For isolation of BM-MSCs, vertebrate discs were irrigated with medium containing 0.05% human serum albumin in phosphate-buffered saline (PBS; Sigma-Aldrich, St. Louis, MO), DNase, and gentamicin (Sigma-Aldrich) and strained through stainless steel sieves. Additional cells were eluted after two rounds of agitation using a prototype bone marrow tumbler. Cells were pooled and filtered through 500- and 200-μm cell strainers (BD Biosciences, San Diego, CA) and centrifuged to recover the pellet. Cells were resuspended and plated out in a T75 flask. Non-adherent cells were washed away after 24 h. When ~90% confluent, cells were frozen.

**Seeding of decellularized scaffolds with LMSCs**

Decellularized lung tissue scaffolds were generated from 3 GOLD stage IV COPD patients with emphysema and 3 non-emphysema controls. Lung tissue blocks (approximately 3 cm^3^) were decellularized using a multi-step, multi-liquid protocol, as previously described[2] and 1 cm^3^ fragments were prepared. These were reseeded with/without 2.5^10^5^ COPD- or control-derived LMSCs in DMEM/F-12 +10% FCS +1% P/S for 24 h with rotation at 37ºC. Reseeded scaffolds were subsequently placed into 24 well plates in DMEM/F-12 + 10% FCS + 1% P/S + 1% amphotericin B and cultured for 1 to 2 weeks, fixed in 4% formalin, embedded in paraffin and sectioned for histological assessment. Paraffin sections were processed and stained with the single chain variable fragment antibody IO3H10 for detection of chondroitin sulfates[3].

**Illumina library prep and sequencing run**

All RNA samples used for library prep had an RNA integrity number (RIN) value above 7.8. Ribosomal RNA was removed by NEXTflex^®^ Poly(A) beads (Bio Scientific) depletion following the manufacturer’s instructions. Purified RNA was then used with the NEXTflex Rapid Directional qRNA-Seq Kits (Bio Scientific) to generate the library according to the manufacturer’s instructions. Briefly, RNA was fragmented, reverse-transcribed to cDNA using random oligonucleotide primers, adenylated and samples ligated to Molecular Index Adapters. The adapter-coupled fragments were extended by PCR to add the sample barcode sequences and the quality of the resulting products was assessed using the Agilent Bioanalyzer DNA 1000 chip microfluidic system (Agilent Technologies). After preparation, the samples were sequenced using the Illumina NextSeq500 Sequencing kit according to the manufacturer’s protocol. Quality control of the raw reads of adapter sequence was done using cutadapt (v2.10). For each sample, the trimmed reads were mapped to the human GRCh37.75 using Burrows-Wheeler Transform (STAR2 v2.5.4) and quantification was determined with HTSeq (v0.11.0).

**Cellular deconvolution**

Cellular deconvolution of bulk RNA-seq data was performed to estimate the proportions of different cell types from the gene expression for all bulk RNA-Seq datasets. This analysis was performed as previously described[4]. Briefly, AutoGeneS software was used on the Human Lung Cell Atlas v1.0 dataset to select and filter 400 genes from highly variable ones. The selection was based on minimized correlation and maximized distance between clusters in which genes with the most stable results across cohorts were selected and used to infer major cell type proportions. The RNA-seq data was subsequently normalized to counts per million (CPM), and highly variable (HV) genes (N=5,000) were selected. Bulk deconvolution on all samples was then conducted using the CIBERSORT support vector regression (SVR) method[5].

**
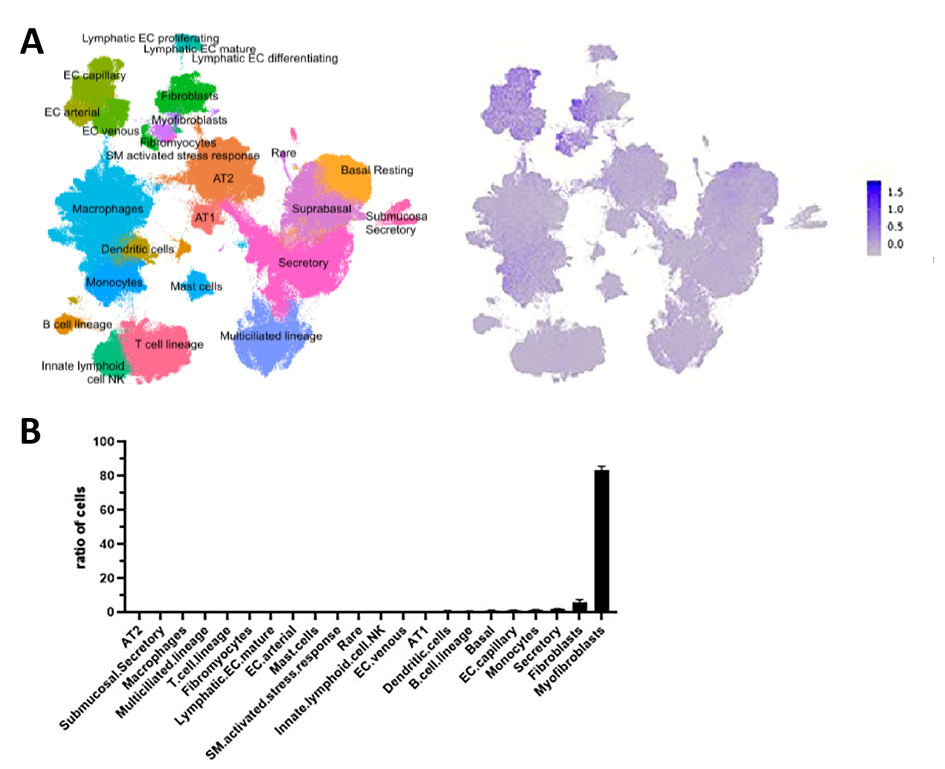
**

**Figure 1. LMSC are associated with myofibroblasts**. A) UMAP in the Human Lung Cell Atlas_V1.0 of the combined expression of unique 5 genes found to be associated with LMSC compared to the MSCs from the other sources (*ETS2*, *TBX5*, *SCN7A*, *FOXF1* and *TBX4*). B) Cellular deconvolution using the single-cell sequencing data from the Human lung cell atlas V1.0 applied to the LMSC RNA-Seq (n=14) using the CIBERSORT method.

**References**

1. Dominici M, Le Blanc K, Mueller I, Slaper-Cortenbach I, Marini F, Krause D, Deans R, Keating A, Prockop D, Horwitz E. Minimal criteria for defining multipotent mesenchymal stromal cells. The International Society for Cellular Therapy position statement. Cytotherapy 2006;8: 315-7 doi:Q2183N8UT042W62H [pii].

2. Kruk DMLW, Wisman M, Bruin HG, Lodewijk ME, Hof DJ, Borghuis T, Daamen WF, van Kuppevelt TH, Timens W, Burgess JK, Ten Hacken NHT, Heijink IH. Abnormalities in reparative function of lung-derived mesenchymal stromal cells in emphysema. Am J Physiol Lung Cell Mol Physiol 2021;320: L832-44 doi:10.1152/ajplung.00147.2020 [doi].

3. Smetsers TF, van de Westerlo EM, ten Dam GB, Overes IM, Schalkwijk J, van Muijen GN, van Kuppevelt TH. Human single-chain antibodies reactive with native chondroitin sulfate detect chondroitin sulfate alterations in melanoma and psoriasis. J Invest Dermatol 2004;122: 707-16 doi:10.1111/j.0022-202X.2004.22316.x [doi].

4. Aliee H, Massip F, Qi C, Stella de Biase M, van Nijnatten J, Kersten ETG, Kermani NZ, Khuder B, Vonk JM, Vermeulen RCH, U-BIOPRED study group, Cambridge Lung Cancer Early Detection Programme, INER-Ciencias Mexican Lung Program, Neighbors M, Tew GW, Grimbaldeston MA, Ten Hacken NHT, Hu S, Guo Y, Zhang X, Sun K, Hiemstra PS, Ponder BA, Makela MJ, Malmstrom K, Rintoul RC, Reyfman PA, Theis FJ, Brandsma CA, Adcock IM, Timens W, Xu CJ, van den Berge M, Schwarz RF, Koppelman GH, Nawijn MC, Faiz A. Determinants of expression of SARS-CoV-2 entry-related genes in upper and lower airways. Allergy 2022;77: 690-4 doi:10.1111/all.15152 [doi].

5. Newman AM, Liu CL, Green MR, Gentles AJ, Feng W, Xu Y, Hoang CD, Diehn M, Alizadeh AA. Robust enumeration of cell subsets from tissue expression profiles. Nat Methods 2015;12: 453-7 doi:10.1038/nmeth.3337 [doi].
